# A molecular palaeobiological exploration of arthropod terrestrialization

**DOI:** 10.1098/rstb.2015.0133

**Published:** 2016-07-19

**Authors:** Jesus Lozano-Fernandez, Robert Carton, Alastair R. Tanner, Mark N. Puttick, Mark Blaxter, Jakob Vinther, Jørgen Olesen, Gonzalo Giribet, Gregory D. Edgecombe, Davide Pisani

**Affiliations:** 1 School of Earth Sciences, University of Bristol, Life Sciences Building, 24 Tyndall Avenue, Bristol BS8 1TQ, UK; 2 School of Biological Sciences, University of Bristol, Life Sciences Building, 24 Tyndall Avenue, Bristol BS8 1TQ, UK; 3 Department of Biology, The National University of Ireland Maynooth, Maynooth, Kildare, Ireland; 4 Institute of Evolutionary Biology, University of Edinburgh, Edinburgh EH9 3TF, UK; 5 Natural History Museum of Denmark, University of Copenhagen, Universitetsparken 15, 2100 Copenhagen, Denmark; 6 Museum of Comparative Zoology, Department of Organismic and Evolutionary Biology, Harvard University, 26 Oxford Street, Cambridge, MA 02138, USA; 7 Department of Earth Sciences, The Natural History Museum, Cromwell Road, London SW7 5BD, UK

**Keywords:** terrestrialization, molecular palaeobiology, arthropod evolution, molecular clock, phylogeny

## Abstract

Understanding animal terrestrialization, the process through which animals colonized the land, is crucial to clarify extant biodiversity and biological adaptation. Arthropoda (insects, spiders, centipedes and their allies) represent the largest majority of terrestrial biodiversity. Here we implemented a molecular palaeobiological approach, merging molecular and fossil evidence, to elucidate the deepest history of the terrestrial arthropods. We focused on the three independent, Palaeozoic arthropod terrestrialization events (those of Myriapoda, Hexapoda and Arachnida) and showed that a marine route to the colonization of land is the most likely scenario. Molecular clock analyses confirmed an origin for the three terrestrial lineages bracketed between the Cambrian and the Silurian. While molecular divergence times for Arachnida are consistent with the fossil record, Myriapoda are inferred to have colonized land earlier, substantially predating trace or body fossil evidence. An estimated origin of myriapods by the Early Cambrian precedes the appearance of embryophytes and perhaps even terrestrial fungi, raising the possibility that terrestrialization had independent origins in crown-group myriapod lineages, consistent with morphological arguments for convergence in tracheal systems.

This article is part of the themed issue ‘Dating species divergences using rocks and clocks’.

## 1. The long road to terrestrial life

Animals and life more broadly have marine origins, and the colonization of land started early in life's history. Possible evidence for subaerial prokaryotic life dates back to the Archaean [1,2], and terrestrial communities (either freshwater or subaerial) with a eukaryotic component are known from the Torridonian of Scotland approximately 1.2–1.0 billion years ago (Gya) [3]. These deposits include multicellular structures, cysts and thalli that can have a diameter of almost 1 mm [3]. While there is no evidence for land plants, animals and fungi, these deposits indicate that at approximately 1 Ga relatively complex terrestrial ecosystems already existed [4]. Definitive evidence for the existence of land plants is much more recent. The oldest embryophyte body fossils are from the Late Silurian [5]. The oldest spores of indisputable embryophyte origin (trilete spores) extend the history of plants only a little deeper, into the Ordovician (449 million years ago—Ma) [4,5], and the oldest embryophyte-like spores (which do not necessarily indicate the existence of embryophytes) barely reach the Late Cambrian [4]. Similarly, the fossil record of the terrestrial Fungi does not extend beyond the Ordovician, with the oldest known fungal fossils dating to approximately 460 Ma [6]. However, terrestrial rock sequences from the Cambrian and the Ediacaran are rare, and the late appearance of land plants and Fungi in the fossil record might represent preservational artefacts of the rock record [4].

Only few animal phyla include lineages that can complete every phase of their life cycle outside of water-saturated environments (from moisture films to the oceans) and are thus fully terrestrial. The most diverse and biologically important of the phyla with lineages that attained full terrestriality are the Vertebrata (with the reptiles, birds and mammals, i.e. Amniota); the Mollusca (with the land snails and the slugs); and the Arthropoda (e.g. insects, spiders, scorpions, centipedes) [7]. While the terrestrial vertebrates colonized the land only once even if some members (such as the cetaceans) secondarily reverted to life in water, molluscs and arthropods colonized the land multiple times independently and at different times in Earth history, constituting better model systems to study terrestrial adaptations at the genomic, physiological and morphological levels. In Arthropoda, there have been a minimum of three ancient (Palaeozoic) terrestrialization events: that of the Hexapoda, that of the Myriapoda and that of the Arachnida [8]. In addition, there have been multiple, more recent, land colonization events within malacostracans. These events correspond to the origin of terrestrial isopods (i.e. the woodlice) and amphipods (e.g. the landhoppers), and of a variety of semi-terrestrial species such as the coconut crab (*Birgus latro*), a decapod that lives its adult life on land but still retains marine larvae (see also [9]).

Previous studies [7,10–13] discussed at length the problems faced by animals crossing the water-to-land barrier, with [11] addressing them specifically in the case of the Arthropoda. These problems mostly relate to the different physical properties of air and water, and affect reproduction, sensory reception, locomotion, gas exchange, osmoregulation and protection from an increased exposure to ultraviolet radiation. A classic example of adaptation to terrestriality at the genomic level is observed, in both vertebrates and arthropods, when comparing the olfactory receptors of marine and terrestrial forms. Terrestrialization is associated with massive, independent, parallel changes in the olfactory receptor gene repertoires of both lineages probably because water-soluble and airborne odorants differ and cannot be efficiently bound by the same receptors [14–16].

Multiple independent terrestrialization events within the same lineage permit rigorous comparison of alternative solutions adopted by different (but genomically and morphophysiologically comparable) groups to the same adaptive challenge, and represent a powerful tool for understanding evolution in a comparative framework [17]. To carry out meaningful comparative studies of animal terrestrialization, however, it is necessary to (i) clarify how many independent terrestrialization events happened in the lineage under scrutiny, (ii) estimate when these terrestrialization events happened and how long they took, and (iii) robustly identify the aquatic sister group of each terrestrial lineage. This information is, in turn, necessary to enable comparative analyses and to estimate the rate at which terrestrial adaptations emerged.

Here we explore the three deepest (Palaeozoic) arthropod terrestrialization events (those of the Hexapoda, Myriapoda and Arachnida), and summarize and expand current evidence about processes that led to their terrestrialization. We particularly focus on Hexapoda, because hexapod terrestrialization, an event that led to the origin of the majority of terrestrial animal biodiversity [18], is particularly poorly understood.

## 2. The phylogenetic perspective

Phylogenetic relationships among the major arthropod lineages have long been debated [19]. However, some consensus has emerged. Myriapoda, the first of the three major terrestrial arthropod groups we shall consider, is now generally accepted to represent the sister group of Pancrustacea (Hexapoda plus all the crustacean lineages). The Myriapoda–Pancrustacea clade is generally referred to as Mandibulata [20–23]. Alternative hypotheses of myriapod relationships have been previously proposed. Among these are the Atelocerata or Tracheata hypothesis, which suggested myriapods as the sister of hexapods, and the Myriochelata hypothesis, which saw the myriapods as the sister group of chelicerates. Atelocerata was based on morphological considerations (e.g. both myriapods and hexapods use tracheae to carry out gas exchange) and continues to have a few adherents among morphologists [24]. However, Atelocerata has only been recovered once in analyses combining molecular, morphological and fossil data [25]. The Myriochelata hypothesis was derived entirely from molecular analyses [26–30], and is now generally considered to have been the result of a long-branch attraction artefact caused by the faster-evolving pancrustaceans attracting to the outgroup and pushing Myriapoda and Chelicerata into an artefactual clade [20]. Both Myriochelata and Atelocerata are disfavoured by current available analyses, with strong molecular and morphological support favouring a placement of hexapods within ‘Crustacea’ (the Pancrustacea or Tetraconata concept—e.g. [20,23,26,31–35]), and a placement of Myriapoda as the sister group of Pancrustacea within Mandibulata (see references above and [19] for a recent review). Accordingly, there is now general agreement that the sister group of the terrestrial Myriapoda is the (primitively) marine Pancrustacea.

The sister group relationships of the Arachnida are quite well understood. This group includes all the terrestrial chelicerates and has two extant successively more distant marine sister taxa: Xiphosura (horseshoe crabs) and Pycnogonida (sea spiders) [23,36,37]. In contrast, the exact relationships of the Hexapoda within Pancrustacea are still unclear, and it is not obvious whether their sister taxon was a marine-, brackish- or freshwater-adapted organism.

Early analyses of eight molecular loci combined with morphological data provided some support for Hexapoda as the sister group of a monophyletic Crustacea, barring a long-branch clade [38], with Branchiopoda as the sister group of Remipedia plus Cephalocarida (the latter two taxa constituting Xenocarida *sensu* [23]). Subsequently, a taxonomically well-sampled molecular phylogeny of three protein coding genes [34] found support for Branchiopoda as the sister group of Hexapoda, and Remipedia as the sister group of those two taxa. While mitogenomic data have also been used in an attempt to resolve hexapod relationships, this type of data is notoriously difficult to analyse [39,40] and has frequently recovered misleading results (contrast [41,42]). With reference to the relationships of Pancrustacea, mitogenomic data were found to be unable to resolve hexapod relationships with confidence [43] and we shall not consider them further.

Based on a large dataset of 62 protein coding genes analysed as nucleotide sequences, support for a sister group relationship between Xenocarida (Remipedia + Cephalocarida—see also above) and Hexapoda was found [23,35]. This clade was called Miracrustacea [23]. In the same analysis, Branchiopoda grouped with Malacostraca, Copepoda and Thecostraca in a novel clade named Vericrustacea [23] rather than allying with Hexapoda. However, these findings were shown to be affected by an artefact of serine codon bias [37]. The close association between Remipedia and Hexapoda (to the exclusion of Cephalocarida) was the only high-level pancrustacean relationship proposed by [23] that was confirmed by [37], which reinstated Branchiopoda as a close relative of Hexapoda, finding Remipedia, Hexapoda, Branchiopoda and Copepoda to constitute an unresolved clade that was referred to as ‘clade A’ in [37]. Other recent studies found similar results, suggesting a Branchiopoda + Hexapoda + Remipedia [21,22,44] (and perhaps Cephalocarida [45]) clade, but with different internal resolutions. In particular, [21,44,45] found Remipedia as the closest relative of Hexapoda (as in [34]), whereas [22] found Branchiopoda as the sister taxon of Hexapoda. Oakley *et al.* [45] was the only one, among the studies mentioned above, that included Cephalocarida, and found Remipedia as the sister group of Hexapoda and Branchiopoda as the sister group of Cephalocarida. Overall, from the perspective of molecular phylogenetics, a strong case can be made that Hexapoda, Branchiopoda and Remipedia belong to the same clade. In addition, evidence exists that Cephalocarida might also be a member of this group of hexapod relatives, which was named Allotriocarida [45]. Yet, to date, molecular phylogenetics has not robustly resolved internal allotriocarid relationships.

A close association between Remipedia and Hexapoda had been suggested based on the presence of a duplication of the haemocyanin gene (haemocyanin being the respiratory pigment used by most arthropods) that is uniquely shared between Remipedia and Hexapoda [46]. This duplication could represent a rare genomic event indicative of a possible sister group relationship between Remipedia and Hexapoda. However, Branchiopoda use haemoglobin as a respiratory pigment rather than haemocyanin. Because haemoglobin is an autapomorphy of Branchiopoda, the presence of two haemocyanin genes in Remipedia and Hexapoda and one in Cephalocarida [46] would conclusively resolve the sister group relationship between these taxa only if the relationships between Cephalocarida and Branchiopoda delineated by [45] were correct. This is because if Cephalocarida (which has only one haemocyanin) is not closely related to Remipedia, Branchiopoda and Hexapoda, then the haemocyanin duplication could have happened in the stem lineage subtending Remipedia, Branchiopoda and Hexapoda, with Branchiopoda having lost both paralogues as it shifted to using haemoglobin as a respiratory pigment. To validate the haemocyanin evidence, it is thus of paramount importance that further studies be carried out to either reject or confirm the results of [45], as bootstrap support values for the monophyly of Allotriocarida and the deepest relationships within this clade were variable and never higher than 85% [45]. Similarities between Remipedia and Hexapoda were also previously suggested based on neurological characters [47,48]. However, more recent studies showed that while neuroanatomical similarities between Hexapoda and Remipedia exist, brain morphology suggests a closer association between Remipedia and Malacostraca [49]. Given that hexapods are generally not found to be close relatives to Malacostraca by other lines of evidence (see above for molecular analyses), similarities in the nervous systems of these three lineages might be subject to evolutionary convergence.

Knowledge of the sister group of each terrestrial arthropod lineage is important not only to increase the power of comparative studies to test adaptive strategies to life on land (see above), but also to understand the route to terrestrialization taken by different lineages. While the sister groups of Myriapoda and Arachnida were undoubtedly marine, most branchiopods inhabit freshwater, and a freshwater route to hexapod terrestrialization was proposed based on this [50]. In contrast, Remipedia is exclusively found in coastal anchialine settings generally with some connection to the sea. Accordingly, a sister group relationship between Remipedia and Hexapoda would better support a direct, marine [10] route to terrestrialization [44].

## 3. The timescale of arthropod terrestrialization

The oldest arthropod fossils are undoubtedly marine. They include trilobites, the oldest representatives of which date back to the Early Cambrian (*ca* 521 Ma [51]); Trilobita is variably interpreted as either stem mandibulates [20] or as stem chelicerates [52]. Other Cambrian, marine fossils include chelicerates (pycnogonids [53]), and crustaceans; both cuticular fragments from Branchiopoda, and possibly also Ostracoda and Copepoda [54] and complete body fossils such as the allotriocarid (most likely stem branchiopod) *Rehbachiella kinnekullensis* [55].

The oldest subaerial arthropod traces (ichnofossils) are from the Mid- to Late Cambrian–Early Ordovician age. Examples include trackways impressed on eolian dune sands by an amphibious myriapod-like arthropod, perhaps a euthycarinoid [56]. Other Cambrian (Mid-Cambrian to Furongian) locomotory traces have been documented from subaerially exposed tidal flats in Wisconsin and Quebec [57]. A euthycarcinoid tracemaker has been confidently associated with these traces, further cementing the view that arthropod subaerial activities (if not terrestrial arthropods) were common on Cambrian shorelines. The oldest terrestrial myriapod body fossil (which is also the oldest undisputably terrestrial animal) is the *ca* 426 Ma millipede *Pneumodesmus newmani,* from the Silurian of Scotland [58]. The subaerial ecology of *P. newmani* is indisputable, because spiracles (segmental openings that allow air to enter the tracheal system) are present on the lateral part of its sternites. The Siluro-Devonian fossil record of Myriapoda consists only of taxa that can be assigned with confidence to the crown groups of extant classes (Diplopoda and Chilopoda), as well as the apparent diplopod-allied Kampecarida, and to date no well corroborated candidates for stem-group Myriapoda have been identified [59]. Critical reviews of the diagnostic/apomorphic characters of myriapods have outlined a search image for a stem-group myriapod that could potentially be recognized in Early Palaeozoic marine strata [60]. Arachnid fossils are just a little younger than those of the oldest Myriapoda, the earliest unequivocally terrestrial examples (trigonotarbids) being present in Silurian deposits dated at approximately 422 Ma [61]. Early Silurian arachnids are represented by the oldest scorpions, which have long been considered to be aquatic because of their associated biota and sediments, but phylogenomic evidence for Scorpiones being nested within terrestrial clades of Arachnida [36] is more compatible with terrestrial habits [62]. The stem group of Arachnida has an aquatic fossil record as far back as the Late Cambrian, the earliest fossils being resting traces of chasmataspidids [63], resolved as sister group to a eurypterid–arachnid clade [64]. Evidence for complex terrestrial ecosystems with land plants, fungi and a variety of arthropods is known from the Upper Silurian onward [65] and is confirmed in the beautifully preserved, and widely celebrated, Lower Devonian (approx. 411 Ma), Rhynie chert Konservat-Lagerstätte [66]. The latter includes the oldest examples of Hexapoda in the fossil record, including Collembola and Insecta.

Recent molecular clock analyses of the arthropod radiation (or of parts of it) generally corroborate the palaeontological evidence and suggest times of origin for Arachnida that are broadly consistent with the fossil evidence [8,21,67–70]. However, molecular divergence times for the origin of crown-group Hexapoda and Myriapoda substantially predate fossils, and this discrepancy is more pronounced in the case of Myriapoda, for which divergence estimates firmly place the modern representatives of this phylum deep in the Cambrian, despite the oldest known crown myriapod fossil being only 426 Ma [58]. This is problematic, because all crown myriapods are terrestrial, and all use tracheae for gas exchange. If tracheae have a single origin in Myriapoda, then current molecular clock results suggest a Cambrian terrestrialization for this lineage, which is not documented in the fossil record. Ephemeral, terrestrial ecosystems existed since approximately 1 Ga [3], and the fossil record of embryophyte-like spores suggests that some form of vegetation existed on land in the Cambrian [2,4,5]. Such limited terrestrial environments, as well as coastal environments [56,57], could have already been conducive to myriapod life on land in the Cambrian [2].

One recent molecular clock study of the arthropod radiation [71], despite being in agreement with other studies with reference to arthropod terrestrialization, is in disagreement with both the fossil record and other molecular clock studies with reference to the deepest divergences in the arthropod tree. However, this study was based on the gene set of [23], that was shown to be affected by strong codon-usage biases [37]. In the absence of correction, this dataset recovered a large number of otherwise unsupported pancrustacean clades (e.g. Vericrustacea and Miracrustacea, see [71]) and consequent erroneous estimation of branch lengths and divergence times. Indeed, subsequent analysis of the same data that attempted to correct for such biases [37] yielded results generally comparable to those obtained in other molecular clock studies.

## 4. A freshwater route to life on land?

An interesting question in the study of terrestrialization is whether land was invaded directly from the sea (the marine route [10,44]), or whether animals first colonized freshwater environments and only subsequently moved to the land (the freshwater route [50]). To address this question, we can look at the fossil record of stem terrestrial lineages when available, and to the sister group of these terrestrial lineages. A freshwater route would imply that the last common ancestor of the considered terrestrial taxa and its sister aquatic lineage separated in a freshwater habitat [50], whereas a marine route would imply that they separated either in a marine or brackish (estuarine) environment [44]. Myriapods and arachnids have marine sister groups. In the case of the Hexapoda, a freshwater route was suggested based on presumed sister-group relationships between Branchiopoda and Hexapoda [50]. While the freshwater origins hypothesis is challenged by the proposal that Remipedia are the sister group of Hexapoda [44], this is far from well established (see above), leaving space for the possibility that hexapod ancestors might have first colonized fresh water and only after that the land. Here we investigate whether hexapods took a marine or a freshwater route to the colonization of land.

## 5. Material and methods

### (a) Dataset assembly

We expanded a published dataset [72] to include new arthropod taxa (see electronic supplementary material, table S1) mostly obtained from NCBI. Transcriptomes of the sea spider *Pycnogonus* sp. and of the horseshoe crab *Limulus polyphemus* were obtained as part of this study and sequenced, respectively, at Edinburgh Genomics and at the Geogenomic Center in Copenhagen. We also added other bilaterian taxa to increase the number of calibration points available for molecular clock analyses (electronic supplementary material, table S1 and figures S1–S5). The core dataset included 57 taxa and 246 genes. This dataset was then pruned of all non-panarthropod species, to avoid systematic biases that might have been induced by the presence of distant outgroups, and create a smaller dataset (including 30 species and 246 genes) used for phylogenetic analyses only. We developed a series of PERL scripts (available at github.com/jairly/MoSuMa_tools) to add species to the existing dataset. BLASTp [73] was used, with an *E*-value cut-off of less than 10^−20^ to identify potential orthologues. The new potential orthologues were aligned with the existing orthologue set using MUSCLE [74], and a maximum-likelihood (ML) tree was generated using PhyML [75] under the LG + G model. Tree distances (branch length distances) were used to distinguish orthologues from paralogues using a few simple rules. (1) If only one putative orthologue existed and its average tree distance from all previously identified orthologues in the dataset was within 3 standard deviations of the average of the tree distances calculated across all previously identified orthologues, then the putative orthologue was retained. (2) If there was only one putative orthologue and its distance to other previously identified orthologues exceeded 3 standard deviations from the average of the tree distances calculated across all previously identified orthologues, then the tree and the alignment were visually inspected. (2a) If the sequence was misaligned, then the alignment was corrected and the procedure repeated. (2b) If the sequence was correctly aligned and the sequence clustered in a phylogenetically unexpected position (e.g. a new *Daphnia* sequence that clustered with a human sequence), then the sequence was deemed a possible paralog and not retained. Note that here ‘phylogenetically unexpected’ simply means obviously incorrect. A myriapod sequence clustering with a chelicerate, for example, was considered to cluster in an expected position, in contrast to *Daphnia* clustering with a human. (2c) If the sequence was correctly aligned and the sequence clustered in a phylogenetically plausible position (e.g. a new *Drosophila* sequence that clustered within insects) the sequence was retained but flagged to allow for directed exclusion (if necessary) in subsequent analyses. (3) If more than one putative orthologue was present in the dataset, then the tree was first visually inspected to evaluate whether all putative orthologues formed a monophyletic group (i.e. to make sure they constituted a set of in-paralogs). (3a) If they did and their average tree distance from other sequences was less than 3 standard deviations from the average distance across all previously identified orthologues, then the putative orthologue of minimal branch length was retained. (3b) If the putative orthologues did not cluster together and all but one had significant distance (in excess of 3 standard deviations) from the average distance across all previously identified orthologues, the putative orthologue of acceptable distance was retained if it also clustered in a phylogenetically plausible position. (3c) If all putative orthologues had excessively long branches (more than 3 standard deviations from the average), then they were all rejected. Each set of orthologues was realigned using MUSCLE [74] and trimmed using Gblocks [76] to exclude ambiguously aligned sections. Gblocks settings were: minimum number of sequences for a conserved position = 50% of the sequences in the protein family; minimum number of sequences for a flank position = 75% of the sequences in the protein family; minimum length of a block = 5; allowed gap positions = half. The final dataset of curated sequences was concatenated using FASconCAT v. 1.0 [77]. It included 58 taxa across all Protostomia and Deuterostomia and 40 657 amino acid positions. Taxa were deleted from this dataset to generate the taxonomically reduced alignment used for phylogenetic reconstruction (see above). The latter included 30 panarthropod species and 40 657 amino acid positions.

### (b) Phylogenetic reconstruction

Phylogenetic trees were inferred using PhyloBayes MPI v. 1.5 [78] under the site-heterogeneous CAT – GTR + G model of amino acid substitution [79]. Convergence was assessed by running two independent Markov chains and using the bpcomp and tracecomp tools from PhyloBayes to monitor the maximum discrepancy in clade support (maxdiff), the effective sample size (effsize) and the relative difference in posterior mean estimates (rel_diff) for several key parameters and summary statistics of the model. The appropriate number of samples to discard as ‘burn in’ was determined first by visual inspection of parameter trace plots, and then by optimizing convergence criteria.

### (c) Molecular clock analyses

Divergence time estimation was performed using PhyloBayes 3.3f (serial version) [80] on a fixed topology (see electronic supplementary material, figures S1–S5). We used two alternative relaxed molecular clock models: the autocorrelated CIR model [81] and the uncorrelated gamma multipliers model (UGAMMA) [82], as in [83]. The tree was rooted on the Deuterostomia–Protostomia split. A set of 24 calibrations (see electronic supplementary material, table S2) was used, with a root prior defined using a Gamma distribution of mean 636 Ma and standard deviation of 30 Ma. However, previously we had also tested the effect of a much more relaxed root prior that used an exponential distribution of average 636 Ma (see electronic supplementary material, table S2 for justifications). The substitution model used to estimate branch lengths was the CAT – GTR + G model, as in the phylogenetic analysis. All analyses were conducted using soft bounds with 5% of the probability mass outside the calibration interval. A birth–death model was used to define prior node ages. Analyses were run under the priors to evaluate the effective joint priors induced by our choice of priors. Convergence was tested running the tracecomp tool as specified above.

### (d) Ancestral environment reconstructions

Maximum-likelihood-based ancestral character state reconstruction was carried in R (www.R-project.org [84]) using maximum-likelihood estimation under the Mk model [85,86] to infer whether the last common ancestor of Branchiopoda was a freshwater-, marine- or brackish-adapted animal. The branchiopod phylogeny of [87] was modified to include key fossils from [88]: *Rehbachiella, Lepidocaris, Castracollis* and *Almatium*. *Rehbachiella kinnekullensis* (from the Upper Cambrian) is particularly important as it was initially described as a marine stem-group anostracan [55], and subsequently reassigned to a stem-group branchiopod [89]. This systematic placement has not been universally accepted, with some analyses instead allying *Rehbachiella* closer to cephalocarids than to branchiopods [45,90]. Whereas *Rehbachiella* is found in association with marine taxa [55], and the geological context of the bituminous limestones in which the fossils are preserved indicates dysoxic marine sediments, most extant branchiopods are found in fresh water or in continental brackish waters (vernal pools, saline lakes, etc.). *Lepidocaris rhyniensis* [91] and *Castracollis wilsonae* [92] are freshwater branchiopod fossils from the Early Devonian Rhynie chert. Kazacharthra (represented herein by *Almatium gusevi* [93]), are Triassic–Jurassic relatives of Notostraca limited to non-marine (lacustrine) deposits from Kazakhstan, Mongolia and China. A matrix representing ecological preferences for all considered taxa was assembled from the literature (see electronic supplementary material, table S3). The time-calibrated tree was generated by adding the fossils from [88] to the tree in [87] using 10 calibrations from [94] and setting tip taxa to their occurrence times. The time-calibrated topology was generated using the R package paleotree [95]. We calculated marginal likelihood under Mk for internal nodes in this time-calibrated tree and present the scaled marginal likelihoods of the three possible root states for total-group Branchiopoda.

## 6. Results

### (a) Phylogeny

**Figure 1.**
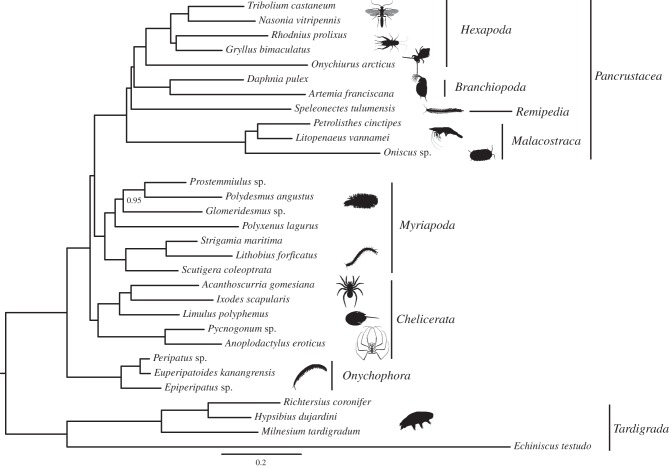
Bayesian phylogeny of Panarthropoda. This tree was obtained under the CAT − GTR + G model. All nodes but one had a posterior probability of 1. bpcomp maxdiff = 0; minimum effective size = 55; maximum rel_diff = 0.2. Most silhouettes from organisms are from Phylopic (phylopic.org/).

Our phylogenetic analyses are presented in figure 1. They clearly support monophyly of Arthropoda and of the three main arthropod lineages (Chelicerata, Myriapoda and Pancrustacea). While a few studies have suggested that Tardigrada, rather than Onychophora, might be the closest sister group of Arthropoda [96], evidence for this phylogenetic arrangement is limited to only a few morphological characters. Our choice of Tardigrada as outgroup is thus guided by results of previous phylogenomic studies [72,97,98]. The relationships among the arthropod lineages are resolved according to current convention and depict a Mandibulata clade (PP = 1) as the sister group of Chelicerata (PP = 1). Within Chelicerata, the sea spiders are recovered as the sister group of the other chelicerates, Euchelicerata (PP = 1), with xiphosurans as sister group to arachnids. Myriapods are likewise well resolved, dividing into Chilopoda and Diplopoda, and each group follows the currently well-accepted relationships [69,99]. Within Pancrustacea, we recovered an arrangement of taxa that is consistent with the monophyly of Allotriocarida. Of particular relevance to terrestrialization is the partial allotriocarid clade, including Branchiopoda, Remipedia and Hexapoda. Within this clade, we found Branchiopoda to be the sister group of Hexapoda (PP = 1), in agreement with [22,37] but contrasting with other studies (as summarized above [21,44,45]).

### (b) Molecular divergence times

**Figure 2.**
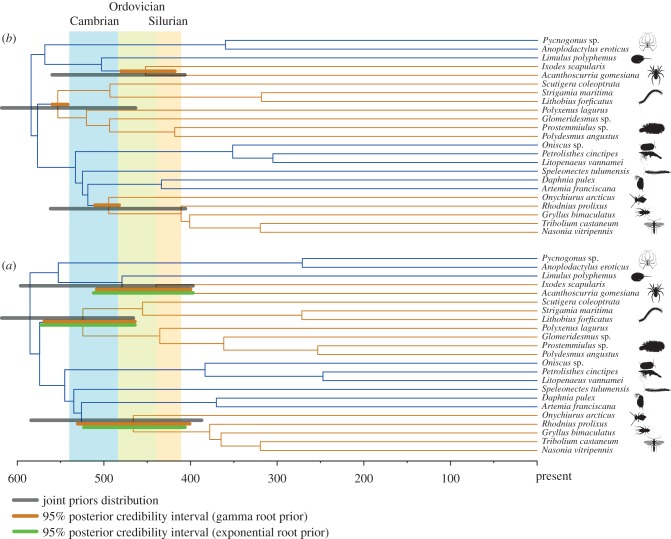
Results of molecular clock analyses. (*a*) Divergence times obtained under the CIR autocorrelated, relaxed, molecular clock model. (*b*) Divergence times obtained using the Uncorrelated Gamma Multipliers model. In both cases, nodes in the tree represent average divergence times estimated using the root prior with 636 Ma mean and 30 Ma SD. Brown bars represent 95% credibility intervals from the considered analysis. Grey bars represent the joint priors (for the considered nodes and analyses). Green bars in figure 2*b* indicate 95% credibility intervals obtained using the exponential prior of average 636 Ma. Blue branches indicate marine lineages. Brown branches terrestrial lineages. In the timescale, numbers represent Myr before the present.

Molecular divergence times among arthropod major clades are presented in figure 2 and table 1 and in electronic supplementary material, figures S1–S5. Results obtained using the UGAMMA model are shown in figure 2*a*, the autocorrelated CIR model in figure 2*b*. Results obtained using the UGAMMA model but with a more permissive exponential root prior are reported in figure 2*a*. Using UGAMMA, 95% credibility intervals surrounding the average divergence times were significantly larger than when the autocorrelated CIR model was used. However, it was evident that for the three nodes of interest (those representing Palaeozoic terrestrialization events) the values in the 95% credibility interval obtained under CIR always represented subsets of the values in the 95% credibility interval obtained using UGAMMA. While the two sets of results are thus statistically indistinguishable, they differ in their congruence with the fossil record. While the more permissive UGAMMA analyses did not reject a Late Cambrian to Silurian origin of the three terrestrial arthropod lineages (the upper limit consistent with the fossil evidence), the CIR model rejected an Ordovician origin for the Myriapoda, suggesting a Precambrian origin instead. Under UGAMMA, arachnid terrestrialization happened in the Silurian, whereas CIR suggests an Ordovician colonization of land. In the case of the Hexapoda, UGAMMA analysis suggested an Ordovician origin, whereas CIR suggested a Cambrian origin and statistically rejected an Early Ordovician origin for this group. Thus, in general, CIR results suggest deeper divergence times. The use of the exponential root, while affecting divergence times of the deepest nodes in our tree (e.g. the age of the Deuterostomia–Protostomia split which is not presented in figure 2, but see electronic supplementary material, figures S1–S5), did not have any effect on the divergence times of the nodes of interest (figure 2 and electronic supplementary material, figure S2).

**Table 1.** Molecular divergence times for key terrestrial arthropod lineages.

taxon	molecular clock model			
	UGAMMA		CIR	
	mean age (Ma)	95% credibility interval	mean age (Ma)	95% credibility interval
Myriapoda	528	568–463	558	572–544
Chilopoda	457	526–408	490	511–452
Diplopoda	439	537–317	519	541–486
Hexapoda	468	512–407	499	431–394
Arachnida	440	518–397	460	493–413

### (c) Ancestral environmental reconstruction

**Figure 3.**
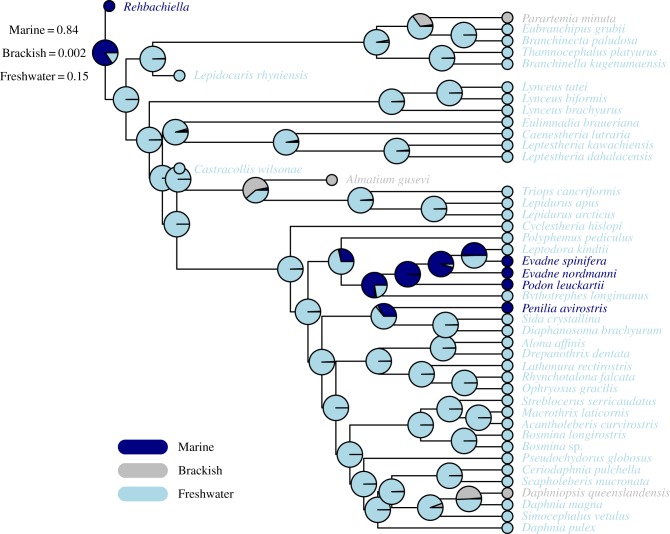
Results of the ancestral environment reconstruction analysis indicating that the last common total-group branchiopod ancestor was most likely a marine organism. The pie charts show the scaled marginal likelihoods of ancestral states for all nodes, with the scaled likelihoods of the total-group ancestor also shown in the text. Branch lengths are proportional to time.

Our ancestral environmental reconstructions (figure 3) aimed to clarify whether the hexapods colonized the land through a freshwater route if their sister group is Branchiopoda rather than Remipedia (figure 1). We found that the last common ancestor of the stem-group Branchiopoda most likely inhabited a marine environment (*p* = 0.84; figure 3). A lower, but not negligible, probability is found for an ancestral freshwater habitat (*p* = 0.15), whereas a brackish ancestry for the total-group Branchiopoda can be confidently rejected (*p* = 0.002; figure 3). Note that these results used a topology where the marine *Rehbachiella* was considered the sister group of the extant branchiopods. As pointed out above, some studies suggested this fossil might instead be allied to cephalocarids [45,90]. If that were the case, given the sister group relationship between cephalocarids and branchiopods suggested in these studies, then a marine origin of Branchiopoda would be inevitable, thus not changing the results of our analyses.

## 7. Discussion

Terrestrialization is the process through which aquatic organisms adapt to a subaerial lifestyle [7], and abundant literature has addressed this process at the physiological level [9,10,12]. However, most of these studies were performed on isolated lineages and did not take full advantage of the comparative approach [17], in part because the application of modern comparative methods [100] needs detailed phylogenetic information and divergence times for terrestrial lineages and their close relatives. Such information has only recently started to be available in sufficient detail.

Our phylogenetic analyses used an expanded multigene dataset of wide systematic scope. While our results are consistent with the monophyly of Allotriocarida, in contrast to [45] and other studies [21,23,35,44], we did not find support for a sister group relationship between Remipedia and Hexapoda. We instead recovered Branchiopoda as the sister group of Hexapoda, as has been proposed previously [22]. Our results cannot be taken as definitive, most importantly because, as with all previous relevant analyses we were able to include only one remipede species, and similar to all previous studies except that of [45], we did not include cephalocarids. With reference to molecular divergence times, whereas [28] obtained the first set of estimates specifically aiming at clarifying terrestrialization in Arthropoda, their study used a dataset composed of only few genes and taxa and molecular clock methods and calibrations that are now obsolete [101]. The most relevant previous molecular clock study specifically addressing arthropod terrestrialization is that of [8], although divergence times among terrestrial lineages can be found in a variety of other studies [21,67–70,102]. Summarizing results from these previous studies indicates that crown (terrestrial) Myriapoda emerged at 554 Ma, crown (terrestrial) Arachnida emerged at 495 Ma, and crown terrestrial Hexapoda emerged at 495 Ma. These divergence times are broadly in line with the results of our analyses (figure 2 and table 1 and electronic supplementary material, figures S1–S5). In the case of Arachnida, this is broadly compatible with the fossil evidence, whereas in the cases of Hexapoda and particularly Myriapoda the molecular divergences are significantly older. Interpretation of the amphibious euthycarcinoids, which first appear in the Cambrian, as stem-group hexapods [103], goes some way to reconciling early estimates for the origin of Hexapoda and the substantially later appearance of crown-group fossils in the Early Devonian.

A recent fossil-independent attempt at dating the metazoan radiation [104] suggested that divergence times that are substantially in line with the fossil record, like all those reported above except [71], represent artefacts caused by over-constrained calibrations, and that the history of animals is much more in line with previous, outdated, findings that suggested the existence of metazoans approximately 1.5 Ga [105]. Indeed, Battistuzzi *et al.* [104] also suggested that the analyses of Wheat & Walberg [71], despite being in strong disagreement with the arthropod fossil record and with other molecular clock studies of the arthropod radiation, may be accurate. As discussed above, however, the results of [71] are based on a dataset affected by strong compositional biases, and used a pancrustacean topology that has now mostly been contradicted. In addition, it has now been shown that there is not enough information left in genomic datasets to correctly estimate rates of evolution in the deepest part of the animal tree without reference to fossils [102], as advocated by Battistuzzi *et al.* [104]. Tellingly, an analysis of the relative rates of substitution per branch inferred by Battistuzzi *et al.* [104] shows them to be identical (and set to the median rate across their entire tree) in 64.5% of the internal branches in their chronogram (electronic supplementary material, figure S6). Furthermore, these constant strict-clock rates are asymmetrically clustered in the root-ward part of their tree. In other words, the relative divergence time approach used in [104] did not relax the clock in the deepest part of their chronogram, and inferred that more than half of opisthokont history (the outgroup in their chronogram is Fungi) was strictly clocklike. The existence of a deep clock for Metazoa and Opisthokonta is clearly unrealistic and is rejected by the data [102], confirming Pisani & Liu's [101] suggestion that relative divergence times cannot meaningfully be applied in deep time. Given the results of [102], and the rate distribution in electronic supplementary material, figure S6, it is not unsurprising that [104] found results comparable to those found in outdated strict-clock studies [105] from two decades ago. From the point of view of arthropod evolution, the convergence of the results of [104] and [71] further suggests that deep divergence times for the origin of Arthropoda are likely to be artefactual.

Considering hexapod terrestrialization, both the freshwater [50] and the marine [44] routes should be considered valid alternatives. Key to distinguishing between the two is understanding whether the last common ancestor of the Hexapoda and either Remipedia or Branchiopoda inhabited a marine, brackish or freshwater habitat. If the last common ancestor of Hexapoda and its sister clade was a freshwater organism, then the colonization of land could have started from a freshwater habitat. If Remipedia (or Remipedia plus Cephalocarida—if Xenocarida were confirmed in future studies) is confirmed as the sister group of Hexapoda, then a marine route would be strongly favoured as there is no evidence that the anchialine–water dwelling remipedes might have ever been living away from the coasts, whereas cephalocarids are marine. If Branchiopoda is confirmed as the sister group of the hexapods, then the situation would be more ambiguous, as modern branchiopods are mostly found in continental waters, leaving the question of the environmental preferences of the last common branchiopod ancestor unresolved. To address this problem, we used ancestral character reconstruction which suggests that, when both extant and fossil taxa are considered, the last common ancestor of Branchiopoda and Hexapoda was most likely a marine organism. Thus, current evidence, when considering phylogenetic uncertainty of hexapod relationships and fossil evidence, seems to favour a marine route to land also for the Hexapoda. Future discoveries of additional Cambrian stem-group branchiopods could better clarify this problem.

## 8. Conclusion

Ephemeral, terrestrial habitats have long existed on the Earth, at the very least since approximately 1 Ga. However, animal terrestrialization was a much more recent process. This was first of all because animals originated in the Cryogenian and radiated close to the base of the Cambrian, in disagreement with [104], and in agreement with [83,102]. Our molecular clock results cannot reject fossil-based divergence times for Arachnida and Hexapoda, and we thus conclude that the most likely scenario, given the current evidence, is that these lineages colonized the land in the Ordovician or the Silurian (Arachnida) and the Ordovician (Hexapoda). Estimates that Myriapoda may have colonized land earlier are in disagreement with the myriapod fossil record, even allowing that terrestrial ecosystems already existed in the Cambrian. A mid-late Cambrian diversification of Diplopoda has, however, been predicted based on geographic distributions of extant millipedes and palaeogeography [106]. We do, however, note that our results for the origins of Chilopoda and Diplopoda are consistent with current fossil evidence (figure 2 and electronic supplementary material, figures S1–S5). One possible scenario that would partly resolve this clash between fossils and molecules would be that these two lineages independently colonized the land; but for that to be the case, tracheae should have evolved independently. This possibility has been suggested previously based on differences in structure of the tracheae and position of the spiracles [107] and should be subjected to critical testing. Irrespective of the precise time at which different arthropods colonized land, it seems currently more likely that the process of animal terrestrialization did not begin before the Late Cambrian and proceeded from the coastline towards the centre of the continents.

## Supplementary Material

Supplementary Information

## Data accessibility

Supplementary Information are available with the paper http://dx.doi.org/10.1098/rstb.2015.0133. The multiple-sequence alignment used for the analyses is available for download at https://bitbucket.org/bzxdp/terrestrialisation_arthropoda.
