# Supplementary material for: A molecular palaeobiological exploration of arthropod terrestrialization
Source: Philos Trans R Soc Lond B Biol Sci. 2016 Jul 19;371(1699):20150133. doi: 10.1098/rstb.2015.0133 (PMC4920334; doi:10.1098/rstb.2015.0133)
Supplement: Supplementary Information [file rstb20150133supp1.pdf]

Supplementary Information
A molecular palaeobiological exploration of arthropod terrestrialisation

Jesus Lozano-Fernandez, Robert Carton, Alastair R. Tanner, Mark N. Puttick, Mark Blaxter, Jakob Vinther, Jørgen Olesen, Gonzalo Giribet, Gregory D. Edgecombe, Davide Pisani

Table S1
Taxa added to Campbell et al. (2011) dataset

	Taxon	Source	Raw Data Phred	N50	Translated Proteins
Remipedia	*Speleonectes tulumensis*	SRR857228	15	774	970
Malacostraca	*Oniscus* sp.	In house	39	363	1,677
Myriapoda	*Prostemmiulus* sp.	SRR945439	39	355	5,849
	Polydesmus angustus	SRR1047642	17	735	5,998
	Glomeridesmus sp.	SRR941771	39	467	25,952
	Polyxenus lagurus	SRR1048056	29	407	1,763
	Lithobius forficatus	SRR1159752	38	227	1,571
	Scutigera coleoptrata	SRR1158078	37	421	43,674
Chelicerata	*Limulus polyphemus*	Geogenomics Copenhagen	37	1181	30,282
	Pycnogonus sp.	Edinburgh Genomics	39	1765	26,668
Tardigrada	*Hypsibius dujardini*	*	*	*	14,421
	Milnesium tardigradum	SRR057381	23-26	1242	18,759
	Echiniscus testudo	SRR1141094	19	790	8,282

*http://badger.bio.ed.ac.uk/H_dujardini/home/download

Table S2
The 24 calibrations used for the molecular clock analysis. These calibrations were taken from: [1–3]

Ixodes - Acanthoscurria	636.1	410
Homo - Danio	444.9	420.7
Aplysia - Lottia	636.1	534
Homo - Gallus	332.9	318
Gallus - Taeniopygia	86	66
Homo - Mus	164.6	61.6
Homo - Xenopus	351	337
Homo - Leucoraja	468.4	420.7
Petromyzon - Homo	636.1	457.5
Ciona - Homo	636.1	514
Strongylocentrosus - Saccoglossus	636.1	515.5
Daphnia - Gryllus	636.1	523
Daphnia - Anoplodactylus	636.1	514
Aplysia - Capitella	636.1	550.25
Epiperipatus - Daphnia	636.1	528.82
Priapulus - Daphnia	636.1	528.82
Anoplodactylus - Acanthoscurria	636.1	497
Scutigera - Strigamia	636.1	413
Scutigera - Glomeridesmus	636.1	419
Rhodnius - Gryllus	414	267
Nasonia - Tribolium	414	307
Nasonia - Onychiurus	636.1	395
Oniscux - Litopenaeus	636.1	358.5

Two alternative root priors were used. The first was a Gamma Distribution with mean = 636 Ma (equivalent to the soft maximum for the Deuterostomia – Protostomia divergence [1], and a standard deviation of 30 Ma. The second root prior tested was an exponential prior of average 636 Ma. This is a much less constrained prior as it would correspond to Gamma distributed priors of average = 636 Ma and standard deviation = 636 Ma.

Table S3

Branchiopod matrix representing ecological preferences for all considered taxa. Species from [4] and [5].

0=marine 1=Brackish 2=Freshwater

Branchinecta paludosa	2
Parartemia minuta	1
Eubranchipus grubii	2
Thamnocephalus platyurus	2
Branchinella kugenumaensis	2
Triops cancriformis	2
Lepidurus arcticus	2
Lepidurus apus	2
Lynceus tatei	2
Lynceus brachyurus	2
Lynceus biformis	2
Leptestheria kawachiensis	2
Leptestheria dahalacensis	2
Eulimnadia braueriana	2
Caenestheria lutraria	2
Cyclestheria hislopi	2
Sida crystallina	2
Diaphanosoma brachyurum	2
Penilia avirostris	0
Polyphemus pediculus	2
Bytho*trephes longimanus*	2
Evadne nordmanni	0
Evadne spinifera	0
Podon leuckartii	0
Leptodora kindtii	2
Daphnia magna	2
Daphnia pulex	2
Ceriodaphnia pulchella	2
Simocephalus vetulus	2
Daphniopsis queenslandensis	1
Scapholeberis mucronata	2
Bosmina longirostris	2
Bosmina sp.	2
Pseudochydorus globosus	2
Alona affinis	2
Rhynchotalona falcata	2
Macrothrix laticornis	2
Lathonura rectirostris	2
Streblocerus serricaudatus	2
Acantholeberis curvirostris	2
Drepanothrix dentata	2
Ophryoxus gracilis	2
Lepidocaris rhyniensis	2
Castracollis wilsonae	2
Almatium gusevi (Kaza.)	1
Rehbachiella	0

Supplementary Figure Captions

Figure S1. Molecular clock analyses under UGAMMA and the Gamma-distributed root prior (average root age =636 Ma; Sd = 30 Ma). Numbers at the nodes are 95% credibility intervals.

Figure S2. Molecular clock analyses under UGAMMA and the exponential root prior of average root age = 636 Ma. This corresponds to a Gamma-distributed prior with average root age = 636 Ma and Sd = 636 Ma. Numbers at the nodes are 95% credibility intervals.

Figure S3. Molecular clock analyses under CIR and the gamma-distributed root prior (average root age = 636 Ma; Sd = 30 Ma). Numbers at the nodes are 95% credibility intervals.

Figure S4. Visualisation of the joint priors in UGAMMA analysis with a gamma-distributed root prior (average root age =636 Ma; Sd = 30 Ma). Numbers at the nodes are 95% credibility intervals.

Figure S5. Visualisation of the joint priors in CIR analysis with a gamma-distributed root prior (average root age = 636 Ma; Sd = 30 Ma). Numbers at the nodes are 95% credibility intervals.

Figure S6. Distribution of relative rates on the tree of [6]. This figure illustrates the failure of non-fossil calibrated molecular clock analyses to relax the clock in the first half of the opisthokont history. Grey depicts internal branches with rate = 1, red depicts internal branches with a rate different from one (rate > 1). Dotted lines terminal branches.

Supplementari References

1. Benton, M. J., Donoghue, P. C. J., Asher, R. J., Friedman, M., Near, T. J. & Vinther, J. 2015 Constraints on the timescale of animal evolutionary history. *Palaeontol. Electronica* **18**, 1–106.

2. Benton, M. J. & Donoghue, P. C. J. 2007 Paleontological evidence to date the tree of life. *Mol. Biol. Evol.* **24**, 26–53.

3. Rota-Stabelli, O., Daley, A. C. & Pisani, D. 2013 Molecular timetrees reveal a cambrian colonization of land and a new scenario for ecdysozoan evolution. *Curr. Biol.* **23**, 392–398.

   4. Stenderup, J. T., Olesen, J. & Glenner, H. 2006 Molecular phylogeny of the Branchiopoda (Crustacea)–Multiple approaches suggest a ‘diplostracan’ ancestry of the Notostraca. *Mol. Phylogenet. Evol.* **41**, 182–194.

   5. Olesen, J. 2007 Monophyly and phylogeny of Branchiopoda, with focus on morphology and homologies of branchiopod phyllopodous limbs. *J. Crustacean Biol.* **27**, 165–183.

4. Battistuzzi, F. U., Billing-Ross, P., Murillo, O., Filipski, A. & Kumar, S. 2015 A Protocol for Diagnosing the Effect of Calibration Priors on Posterior Time Estimates: A Case Study for the Cambrian Explosion of Animal Phyla. *Mol. Biol. Evol.* **32**, 1907–1912.

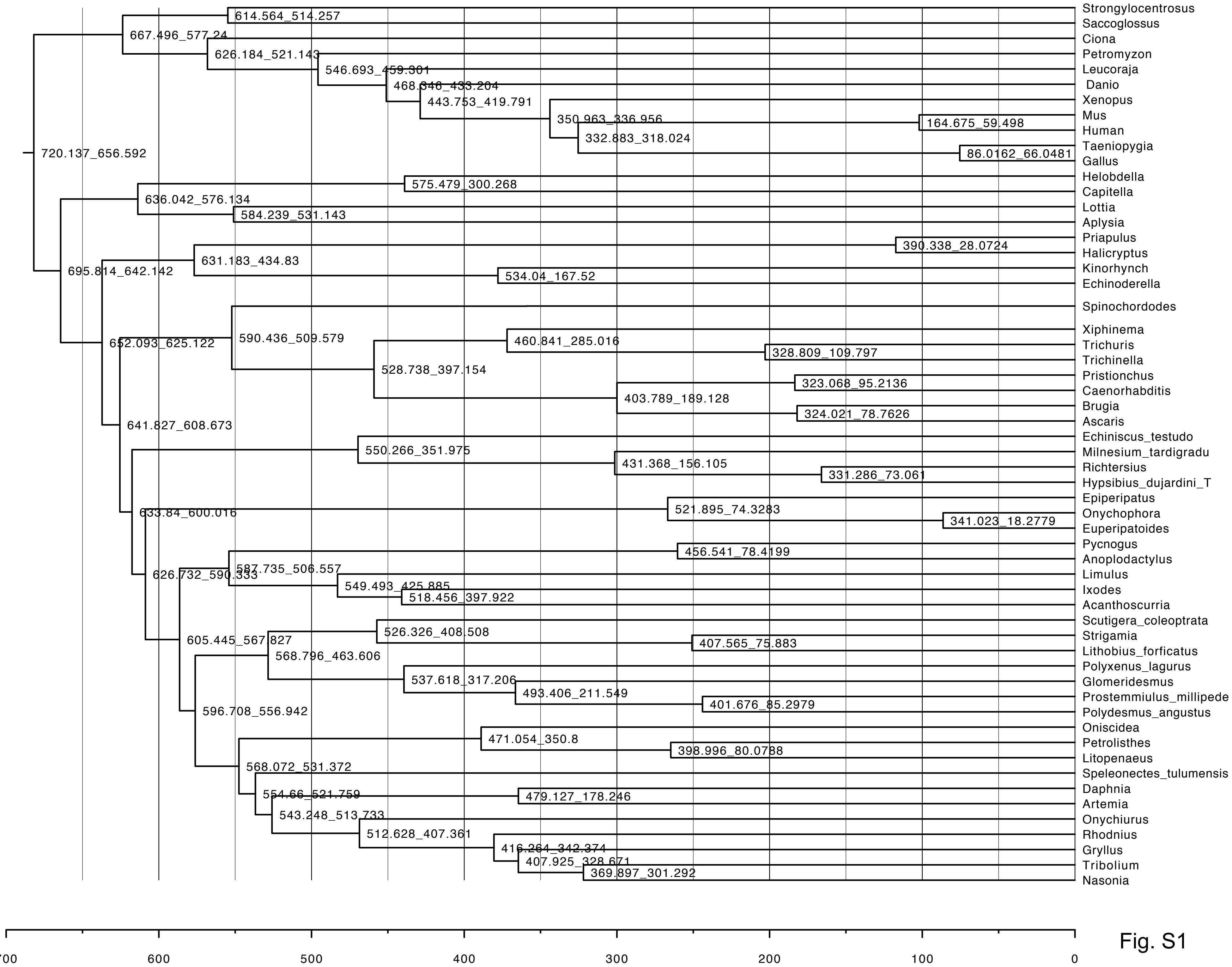

Strongylocentrosus
Saccoglossus
Ciona
Petromyzon
Leucoraja
Danio
Xenopus
Mus
Human
Taeniopygia
Gallus
Helobdella
Capitella
Lottia
Aplysia
Priapulus
Halicryptus
Kinorhynch
Echinoderella
Spinochordodes
Xiphinema
Trichuris
Trichinella
Pristionchus
Caenorhabditis
Brugia
Ascaris
Echiniscus_testudo
Milnesium_tardigradu
Richtersius
Hypsibius_dujardini_T
Epiperipatus
Onychophora
Euperipatoides
Pycnogus
Anoplodactylus
Limulus
Ixodes
Acanthoscurria
Scutigera_coleoptrata
Strigamia
Lithobius_forficatus
Polyxenus_lagurus
Glomeridesmus
Prostemmiulus_millipede
Polydesmus_angustus
Oniscidea
Petrolisthes
Litopenaeus
Speleonectes_tulumensis
Daphnia
Artemia
Onychiurus
Rhodnius
Gryllus
Tribolium
Nasonia
720.137_656.592
667.496_577.24
614.564_514.257
626.184_521.143
546.693_459.301
468.346_433.204
443.753_419.791
350.963_336.956
332.883_318.024
164.675_59.498
86.0162_66.0481
695.814_642.142
636.042_576.134
575.479_300.268
584.239_531.143
631.183_434.83
390.338_28.0724
534.04_167.52
652.093_625.122
590.436_509.579
460.841_285.016
328.809_109.797
528.738_397.154
323.068_95.2136
403.789_189.128
324.021_78.7626
641.827_608.673
550.266_351.975
431.368_156.105
331.286_73.061
633.84_600.016
521.895_74.3283
341.023_18.2779
456.541_78.4199
587.735_506.557
626.732_590.333
549.493_425.885
518.456_397.922
526.326_408.508
605.445_567.827
407.565_75.883
568.796_463.606
537.618_317.206
493.406_211.549
401.676_85.2979
596.708_556.942
471.054_350.8
398.996_80.0788
568.072_531.372
554.66_521.759
479.127_178.246
543.248_513.733
512.628_407.361
416.264_342.374
407.925_328.671
369.897_301.292
700
600
500
400
300
200
100
0

Fig. S1

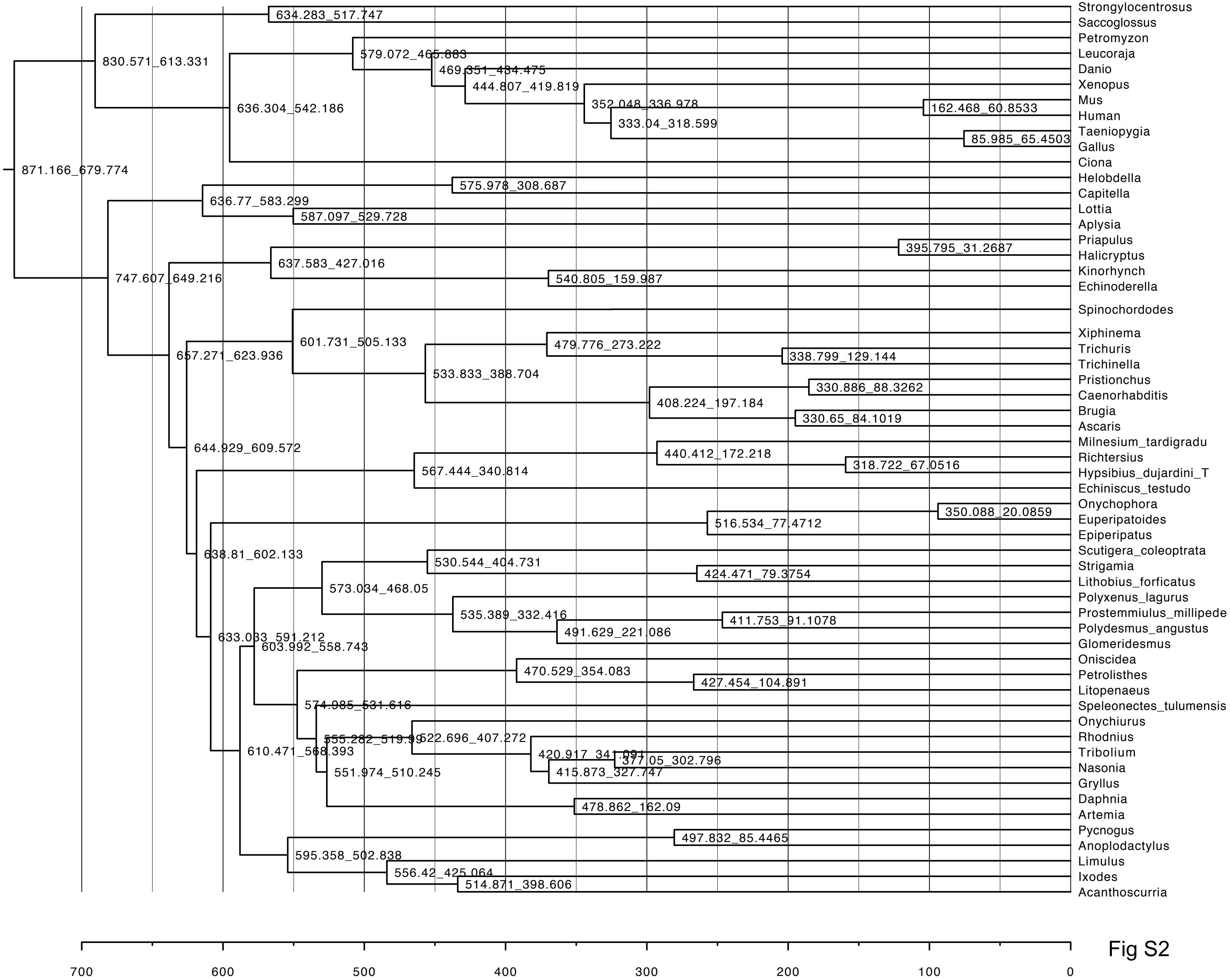
Strongylocentrosus
Saccoglossus
Petromyzon
Leucoraja
Danio
Xenopus
Mus
Human
Taeniopygia
Gallus
Ciona
Helobdella
Capitella
Lottia
Aplysia
Priapulus
Halicryptus
Kinorhynch
Echinoderella
Spinochordodes
Xiphinema
Trichuris
Trichinella
Pristionchus
Caenorhabditis
Brugia
Ascaris
Milnesium_tardigradu
Richtersius
Hypsibius_dujardini_T
Echiniscus_testudo
Onychophora
Euperipatoides
Epiperipatus
Scutigera_coleoptrata
Strigamia
Lithobius_forficatus
Polyxenus_lagurus
Prostemmiulus_millipede
Polydesmus_angustus
Glomeridesmus
Oniscidea
Petrolisthes
Litopenaeus
Speleonectes_tulumensis
Onychiurus
Rhodnius
Tribolium
Nasonia
Gryllus
Daphnia
Artemia
Pycnogus
Anoplodactylus
Limulus
Ixodes
Acanthoscurria
871.166_679.774
830.571_613.331
634.283_517.747
636.304_542.186
579.072_465.883
469.351_434.475
444.807_419.819
352.048_336.978
333.04_318.599
162.468_60.8533
85.985_65.4503
747.607_649.216
636.77_583.299
575.978_308.687
587.097_529.728
637.583_427.016
395.795_31.2687
540.805_159.987
657.271_623.936
601.731_505.133
479.776_273.222
338.799_129.144
533.833_388.704
330.886_88.3262
408.224_197.184
330.65_84.1019
644.929_609.572
440.412_172.218
567.444_340.814
318.722_67.0516
350.088_20.0859
516.534_77.4712
638.81_602.133
530.544_404.731
424.471_79.3754
573.034_468.05
535.389_332.416
411.753_91.1078
491.629_221.086
633.033_591.212
603.992_558.743
470.529_354.083
427.454_104.891
574.985_531.616
555.282_519.99
522.696_407.272
420.917_341.091
377.05_302.796
415.873_327.747
610.471_568.393
551.974_510.245
478.862_162.09
497.832_85.4465
595.358_502.838
556.42_425.064
514.871_398.606
700
600
500
400
300
200
100
0

Fig S2

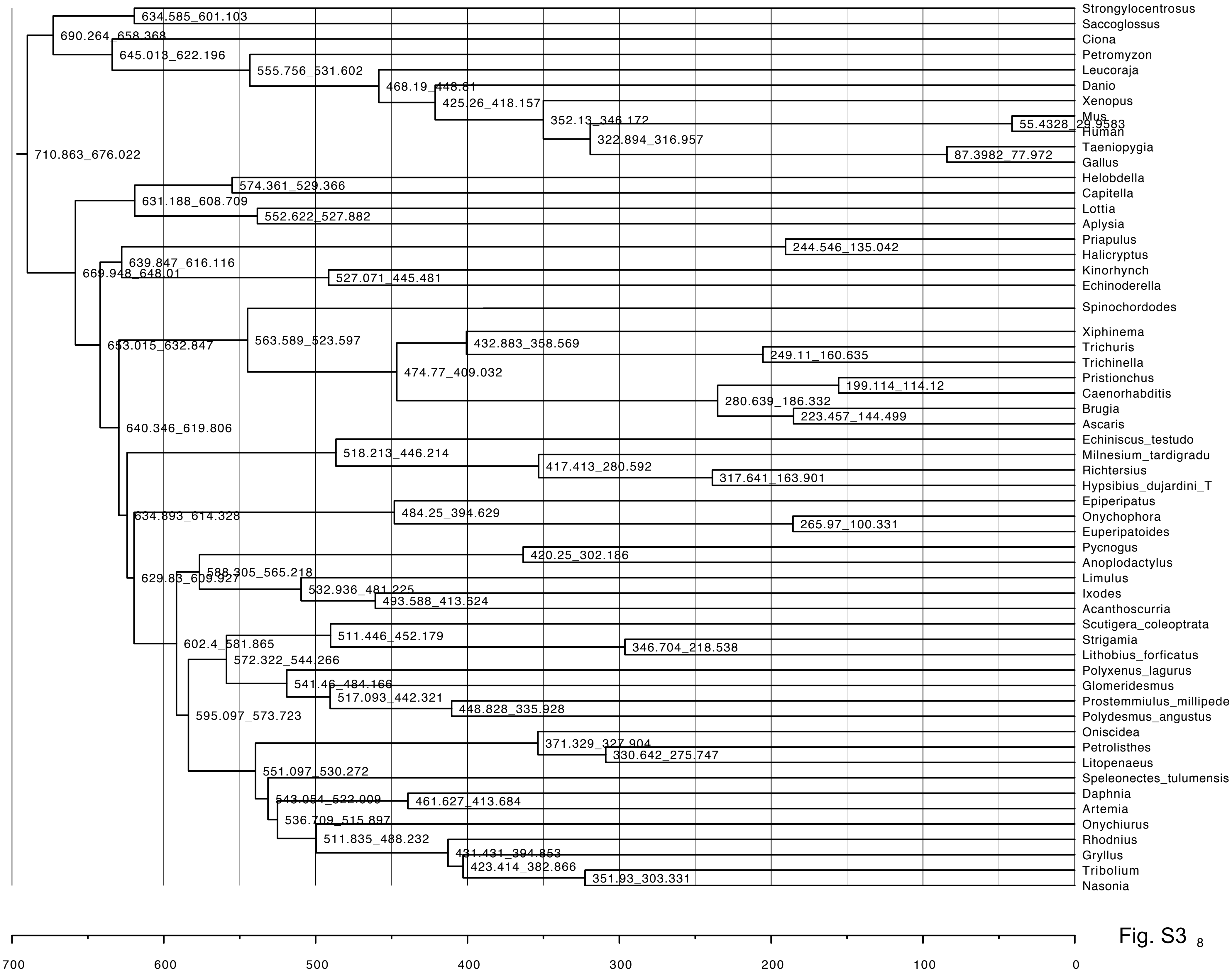

Strongylocentrosus
Saccoglossus
Ciona
Petromyzon
Leucoraja
Danio
Xenopus
Mus
Human
Taeniopygia
Gallus
Helobdella
Capitella
Lottia
Aplysia
Priapulus
Halicryptus
Kinorhynch
Echinoderella
Spinochordodes
Xiphinema
Trichuris
Trichinella
Pristionchus
Caenorhabditis
Brugia
Ascaris
Echiniscus_testudo
Milnesium_tardigradu
Richtersius
Hypsibius_dujardini_T
Epiperipatus
Onychophora
Euperipatoides
Pycnogus
Anoplodactylus
Limulus
Ixodes
Acanthoscurria
Scutigera_coleoptrata
Strigamia
Lithobius_forficatus
Polyxenus_lagurus
Glomeridesmus
Prostemmiulus_millipede
Polydesmus_angustus
Oniscidea
Petrolisthes
Litopenaeus
Speleonectes_tulumensis
Daphnia
Artemia
Onychiurus
Rhodnius
Gryllus
Tribolium
Nasonia
710.863_676.022
690.264_658.368
634.585_601.103
645.013_622.196
555.756_531.602
468.19_448.81
425.26_418.157
352.13_346.172
322.894_316.957
55.4328_29.9583
87.3982_77.972
669.948_648.01
631.188_608.709
574.361_529.366
552.622_527.882
639.847_616.116
244.546_135.042
527.071_445.481
653.015_632.847
563.589_523.597
474.77_409.032
432.883_358.569
249.11_160.635
280.639_186.332
199.114_114.12
223.457_144.499
640.346_619.806
518.213_446.214
417.413_280.592
317.641_163.901
634.893_614.328
484.25_394.629
265.97_100.331
629.83_609.927
588.305_565.218
420.25_302.186
532.936_481.225
493.588_413.624
602.4_581.865
572.322_544.266
511.446_452.179
346.704_218.538
541.46_484.166
517.093_442.321
448.828_335.928
595.097_573.723
551.097_530.272
371.329_327.904
330.642_275.747
543.054_522.009
461.627_413.684
536.709_515.897
511.835_488.232
431.431_394.853
423.414_382.866
351.93_303.331
700
600
500
400
300
200
100
0

Fig. S3

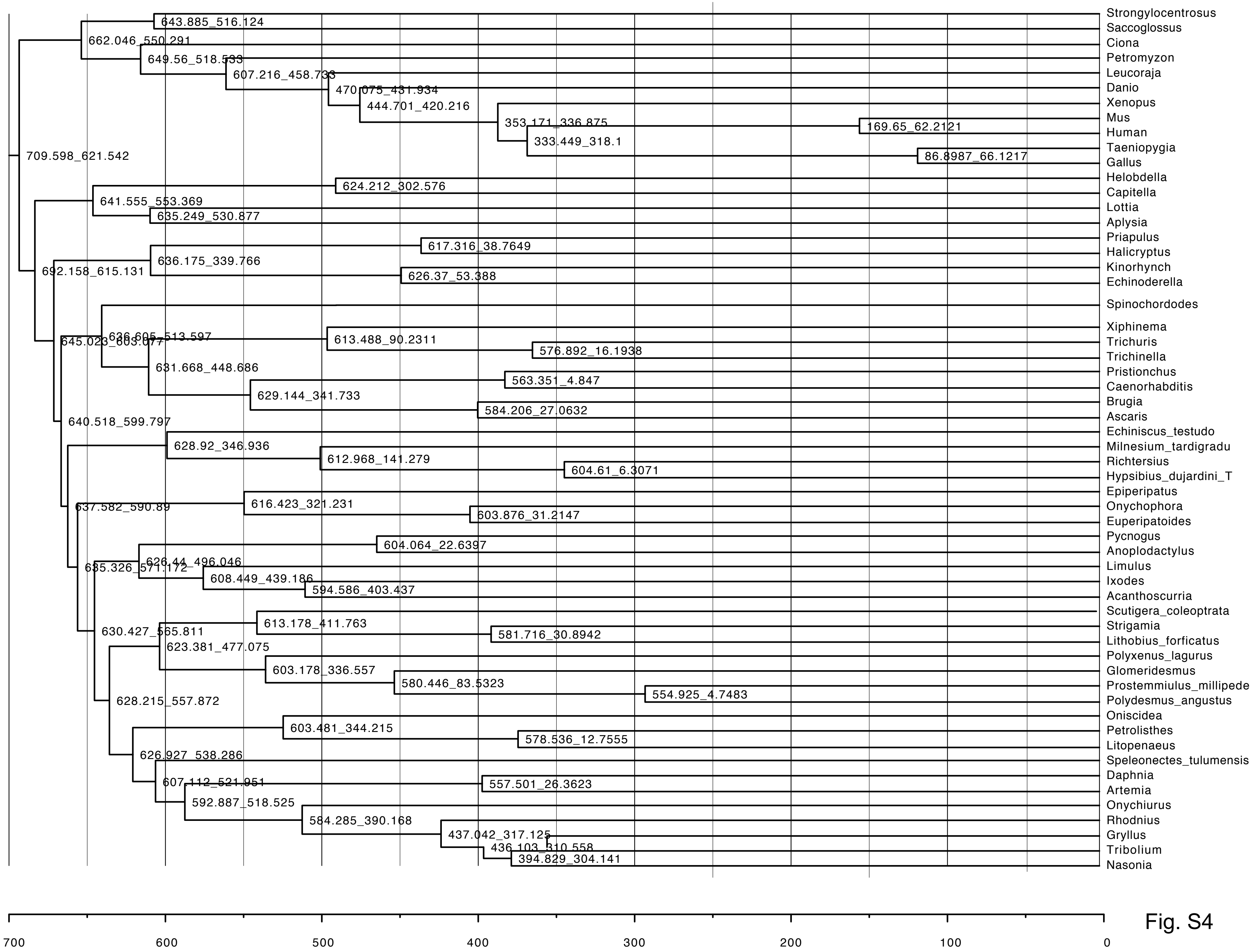
Strongylocentrosus
Saccoglossus
Ciona
Petromyzon
Leucoraja
Danio
Xenopus
Mus
Human
Taeniopygia
Gallus
Helobdella
Capitella
Lottia
Aplysia
Priapulus
Halicryptus
Kinorhynch
Echinoderella
Spinochordodes
Xiphinema
Trichuris
Trichinella
Pristionchus
Caenorhabditis
Brugia
Ascaris
Echiniscus_testudo
Milnesium_tardigradu
Richtersius
Hypsibius_dujardini_T
Epiperipatus
Onychophora
Euperipatoides
Pycnogus
Anoplodactylus
Limulus
Ixodes
Acanthoscurria
Scutigera_coleoptrata
Strigamia
Lithobius_forficatus
Polyxenus_lagurus
Glomeridesmus
Prostemmiulus_millipede
Polydesmus_angustus
Oniscidea
Petrolisthes
Litopenaeus
Speleonectes_tulumensis
Daphnia
Artemia
Onychiurus
Rhodnius
Gryllus
Tribolium
Nasonia
643.885_516.124
662.046_550.291
649.56_518.533
607.216_458.733
470.075_431.934
444.701_420.216
353.171_336.875
169.65_62.2121
333.449_318.1
86.8987_66.1217
709.598_621.542
624.212_302.576
641.555_553.369
635.249_530.877
617.316_38.7649
636.175_339.766
692.158_615.131
626.37_53.388
636.605_513.597
645.023_603.077
613.488_90.2311
576.892_16.1938
631.668_448.686
563.351_4.847
629.144_341.733
584.206_27.0632
640.518_599.797
628.92_346.936
612.968_141.279
604.61_6.3071
616.423_321.231
637.582_590.89
603.876_31.2147
604.064_22.6397
626.44_496.046
635.326_571.172
608.449_439.186
594.586_403.437
613.178_411.763
630.427_565.811
581.716_30.8942
623.381_477.075
603.178_336.557
580.446_83.5323
554.925_4.7483
628.215_557.872
603.481_344.215
578.536_12.7555
626.927_538.286
607.112_521.951
557.501_26.3623
592.887_518.525
584.285_390.168
437.042_317.125
436.103_310.558
394.829_304.141
700
600
500
400
300
200
100
0

Fig. S4

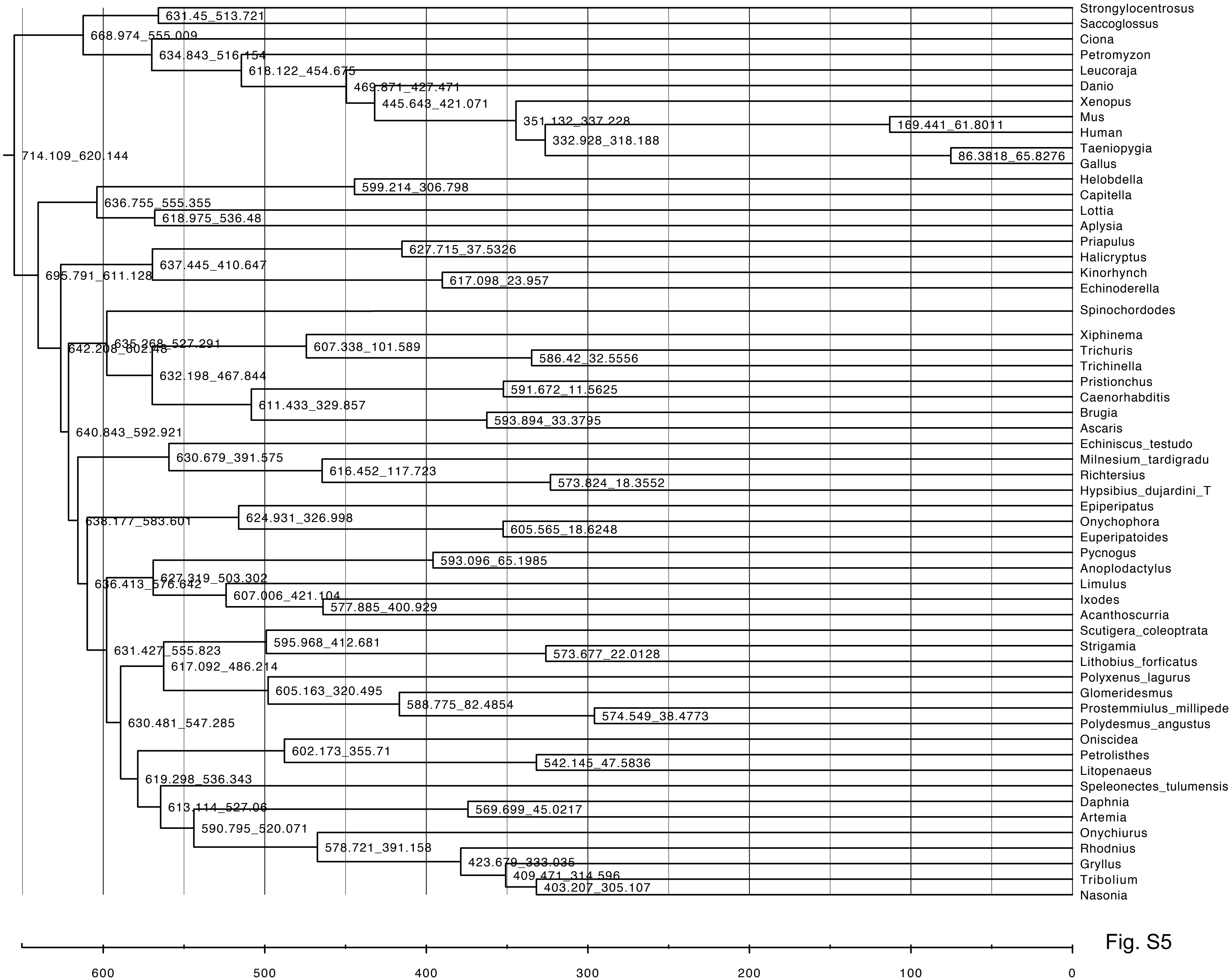

Strongylocentrosus
Saccoglossus
Ciona
Petromyzon
Leucoraja
Danio
Xenopus
Mus
Human
Taeniopygia
Gallus
Helobdella
Capitella
Lottia
Aplysia
Priapulus
Halicryptus
Kinorhynch
Echinoderella
Spinochordodes
Xiphinema
Trichuris
Trichinella
Pristionchus
Caenorhabditis
Brugia
Ascaris
Echiniscus_testudo
Milnesium_tardigradu
Richtersius
Hypsibius_dujardini_T
Epiperipatus
Onychophora
Euperipatoides
Pycnogus
Anoplodactylus
Limulus
Ixodes
Acanthoscurria
Scutigera_coleoptrata
Strigamia
Lithobius_forficatus
Polyxenus_lagurus
Glomeridesmus
Prostemmiulus_millipede
Polydesmus_angustus
Oniscidea
Petrolisthes
Litopenaeus
Speleonectes_tulumensis
Daphnia
Artemia
Onychiurus
Rhodnius
Gryllus
Tribolium
Nasonia
714.109_620.144
668.974_555.009
631.45_513.721
634.843_516.154
618.122_454.675
469.871_427.471
445.643_421.071
351.132_337.228
332.928_318.188
169.441_61.8011
86.3818_65.8276
695.791_611.128
636.755_555.355
599.214_306.798
618.975_536.48
637.445_410.647
627.715_37.5326
617.098_23.957
642.208_602.48
635.268_527.291
607.338_101.589
586.42_32.5556
632.198_467.844
591.672_11.5625
611.433_329.857
593.894_33.3795
640.843_592.921
630.679_391.575
616.452_117.723
573.824_18.3552
638.177_583.601
624.931_326.998
605.565_18.6248
636.413_576.642
593.096_65.1985
627.319_503.302
607.006_421.104
577.885_400.929
631.427_555.823
595.968_412.681
573.677_22.0128
617.092_486.214
605.163_320.495
588.775_82.4854
574.549_38.4773
630.481_547.285
602.173_355.71
542.145_47.5836
619.298_536.343
613.114_527.06
569.699_45.0217
590.795_520.071
578.721_391.158
423.679_333.035
409.471_314.596
403.207_305.107
600
500
400
300
200
100
0

Fig. S5

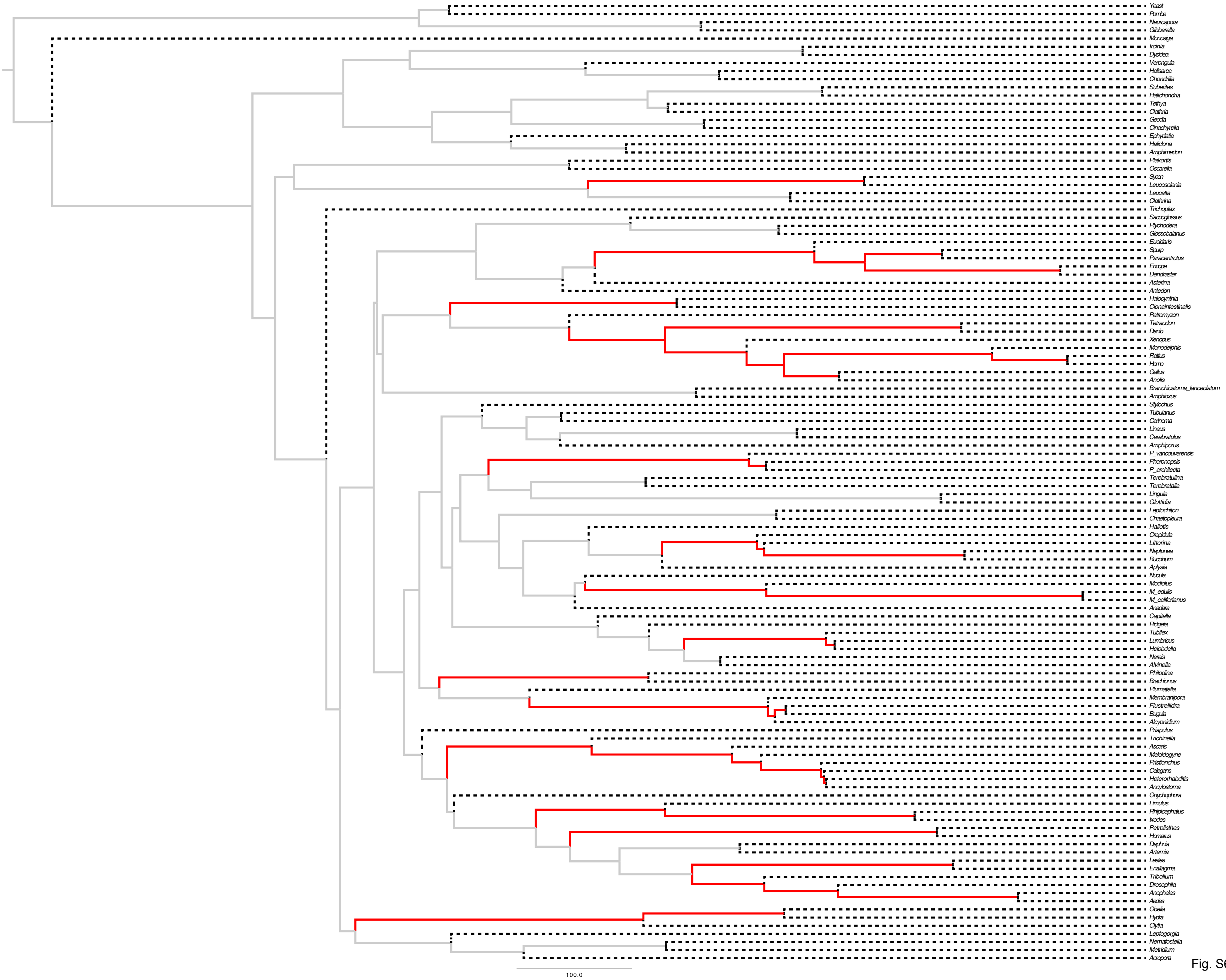
Yeast
Pombe
Neurospora
Gibberella
Monosiga
Ircinia
Dysidea
Verongula
Halisarca
Chondrilla
Suberites
Halichondria
Tethya
Clathria
Geodia
Cinachyrella
Ephydatia
Haliclona
Amphimedon
Plakortis
Oscarella
Sycon
Leucosolenia
Leucetta
Clathrina
Trichoplax
Saccoglossus
Ptychodera
Glossobalanus
Eucidaris
Spurp
Paracentrotus
Encope
Dendraster
Asterina
Antedon
Halocynthia
Cionaintestinalis
Petromyzon
Tetraodon
Danio
Xenopus
Monodelphis
Rattus
Homo
Gallus
Anolis
Branchiostoma_lanceolatum
Amphioxus
Stylochus
Tubulanus
Carinoma
Lineus
Cerebratulus
Amphiporus
P_vancouverensis
Phoronopsis
P_architecta
Terebratulina
Terebratalia
Lingula
Glottidia
Leptochiton
Chaetopleura
Haliotis
Crepidula
Littorina
Neptunea
Buccinum
Aplysia
Nucula
Modiolus
M_edulis
M_californianus
Anadara
Capitella
Ridgeia
Tubifex
Lumbricus
Helobdella
Nereis
Alvinella
Philodina
Brachionus
Plumatella
Membranipora
Flustrellidra
Bugula
Alcyonidium
Priapulus
Trichinella
Ascaris
Meloidogyne
Pristionchus
Celegans
Heterorhabditis
Ancylostoma
Onychophora
Limulus
Rhipicephalus
Ixodes
Petrolisthes
Homarus
Daphnia
Artemia
Lestes
Enallagma
Tribolium
Drosophila
Anopheles
Aedes
Obelia
Hydra
Clytia
Leptogorgia
Nematostella
Metridium
Acropora
100.0

Fig. S6
